# Supplementary material for: Four Unique Genetic Variants in Three Genes Account for 62.7% of Early-Onset Severe Retinal Dystrophy in Chile: Diagnostic and Therapeutic Consequences
Source: Int J Mol Sci. 2024 Jun 3;25(11):6151. doi: 10.3390/ijms25116151 (PMC11172861; doi:10.3390/ijms25116151)
Supplement: Supplementary file 1 [file ijms-25-06151-s001.zip › Tables/Supplementary Tables S1 LFT.pdf]

Gene	Transcript ID (RefSeq)	Transcript ID (Ensembl)
ABCA4	NM_000350	**ENST00000370225**
ABCA4	NM_000350	**ENST00000370225**
ABCA4	NM_000350	**ENST00000370225**
ABHD12	NM_015600	**ENST00000376542**
ABHD12	NM_001042472	**ENST00000339157**
ABHD12	NM_015600	**ENST00000376542**
ABHD12	NM_001042472	**ENST00000339157**
ACBD5	NM_145698	**ENST00000396271**
ACBD5	NM_001352568	**ENST00000426079**
ACBD5	NM_001352571	**ENST00000375888**
ADAM9	NM_003816	**ENST00000487273**
ADAMTS18	NM_199355	**ENST00000282849**
ADAMTS18	NM_199355	**ENST00000282849**
ADIPOR1	NM_015999	**ENST00000340990**
AGBL5	NM_021831	**ENST00000360131**
AGBL5	NM_001035507	**ENST00000323064**
AHI1	NM_017651	**ENST00000457866**
AHI1	NM_001134832	**ENST00000327035**
AHI1	NM_017651	**ENST00000457866**
AHI1	NM_001134832	**ENST00000327035**
AIPL1	NM_014336	**ENST00000381129**
AIPL1	NM_001285403	**ENST00000571740**
AIPL1	NM_014336	**ENST00000381129**
AIPL1	NM_001285403	**ENST00000571740**
ALMS1	NM_015120	**ENST00000264448**
ALMS1	NM_015120	**ENST00000264448**
ALMS1	NM_015120	**ENST00000264448**
ARL13B	NM_182896	**ENST00000471138**
ARL2BP	NM_012106	**ENST00000219204**
ARL6	NM_032146	**ENST00000493990**
ATF6	NM_007348	**ENST00000367942**
ATF6	NM_007348	**ENST00000367942**
ATF6	NM_007348	**ENST00000367942**
ATOH7	NM_145178	**ENST00000373673**
ATXN7	NM_001177387	**ENST00000538065**
ATXN7	NM_001128149	**ENST00000484332**
BBS1	NM_024649	**ENST00000318312**
BBS1	NM_024649	**ENST00000318312**
BBS12	NM_152618	**ENST00000314218**
BBS2	NM_031885	**ENST00000245157**
BBS5	NM_152384	**ENST00000295240**
BBS5	NM_152384	**ENST00000295240**
BEST1	NM_001139443	**ENST00000449131**
BEST1	NM_004183	**ENST00000378043**
BEST1	NM_001139443	**ENST00000449131**
BEST1	NM_004183	**ENST00000378043**
BEST1	NM_001139443	**ENST00000449131**
BEST1	NM_004183	**ENST00000378043**
BMP4	NM_001202	**ENST00000245451**
C1QTNF5	NM_001278431	**ENST00000528368**
C21ORF2	NM_001271441	**ENST00000397956**
C21ORF2	NM_001271441	**ENST00000397956**
C21ORF2	NM_001271441	**ENST00000397956**

C2ORF71	NM_001029883	**ENST00000331664**
C2ORF71	NM_001029883	**ENST00000331664**
C2ORF71	NM_001029883	**ENST00000331664**
C5ORF42	NM_023073.3	**ENST00000425232**
C8ORF37	NM_177965	**ENST00000286688**
C8ORF37	NM_177965.5	**ENST00000286688**
C8ORF37	NM_177965	**ENST00000286688**
CA4	NM_000717	**ENST00000300900**
CABP4	NM_145200	**ENST00000325656**
CABP4	NM_001300896	**ENST00000438189**
CABP4	NM_145200	**ENST00000325656**
CABP4	NM_001300896	**ENST00000438189**
CABP4	NM_145200	**ENST00000325656**
CABP4	NM_001300896	**ENST00000438189**
CACNA1F	NM_005183	**ENST00000376265**
CACNA1F	NM_001256790	**ENST00000376251**
CACNA1F	NM_005183	**ENST00000376265**
CACNA1F	NM_001256790	**ENST00000376251**
CACNA1F	NM_005183	**ENST00000376265**
CACNA1F	NM_001256790	**ENST00000376251**
CACNA1F	NM_005183	**ENST00000376265**
CACNA1F	NM_001256790	**ENST00000376251**
CACNA2D4	NM_172364	**ENST00000382722**
CAPN5	NM_004055	**ENST00000529629**
CC2D2A	NM_001080522	**ENST00000413206**
CC2D2A	NM_020785	**ENST00000503658**
CC2D2A	NM_001164720	**ENST00000515124**
CC2D2A	NM_001080522	**ENST00000413206**
CC2D2A	NM_020785	**ENST00000503658**
CC2D2A	NM_001164720	**ENST00000515124**
CCT2	NM_006431	**ENST00000299300**
CCT2	NM_006431	**ENST00000299300**
CDH3	NM_001793	**ENST00000264012**
CDH3	NM_001317195	**ENST00000429102**
CDHR1	NM_033100	**ENST00000372117**
CDHR1	NM_001171971	**ENST00000332904**
CEP104	NM_014704	**ENST00000378230**
CEP164	NM_014956	**ENST00000278935**
CEP250	NM_007186	**ENST00000397527**
CEP290	NM_025114	**ENST00000552810**
CEP290	NM_025114	**ENST00000552810**
CEP290	NM_025114	**ENST00000552810**
CEP41	NM_018718	**ENST00000223208**
CEP41	NM_001257160	**ENST00000489512**
CEP78	NM_001098802	**ENST00000376597**
CEP78	NM_001330691	**ENST00000376598**
CEP78	NM_001098802	**ENST00000376597**
CEP78	NM_001330691	**ENST00000376598**
CERKL	NM_001030311	**ENST00000339098**
CERKL	NM_001030311	**ENST00000339098**
CHM	NM_000390	**ENST00000357749**
CHM	NM_001145414	**ENST00000358786**
CHM	NM_000390	**ENST00000357749**
CHM	NM_001145414	**ENST00000358786**
CLN1/PPT1	NM_000310	**ENST00000433473**

CLN2/TPP1	NM_000391	**ENST00000299427**
CLN3	NM_000086	**ENST00000359984**
CLN3	NM_000086	**ENST00000359984**
CLRN1	NM_001195794	**ENST00000328863**
CLRN1	NM_052995	**ENST00000295911**
CLUAP1	NM_015041	**ENST00000576634**
CLUAP1	NM_015041	**ENST00000576634**
CLUAP1	NM_015041	**ENST00000576634**
CNGA1	NM_001142564	**ENST00000402813**
CNGA3	NM_001298	**ENST00000393504**
CNGA3	NM_001298	**ENST00000393504**
CNGA3	NM_001298	**ENST00000393504**
CNGA3	NM_001298	**ENST00000393504**
CNGA3	NM_001298	**ENST00000393504**
CNGB1	NM_001297	**ENST00000251102**
CNGB1	NM_001135639	**ENST00000311183**
CNGB3	NM_019098	**ENST00000320005**
CNGB3	NM_019098	**ENST00000320005**
CNGB3	NM_019098	**ENST00000320005**
CNGB3	NM_019098	**ENST00000320005**
CNNM4	NM_020184	**ENST00000377075**
COL11A1	NM_080629	**ENST00000358392**
COL11A1	NM_001854	**ENST00000370096**
COL18A1	NM_030582	**ENST00000355480**
COL18A1	NM_130445	**ENST00000400337**
COL18A1	NM_130444	**ENST00000359759**
COL2A1	NM_001844	**ENST00000380518**
CRB1	NM_201253	**ENST00000367400**
CRB1	NM_001257965	**ENST00000535699**
CRB1	NM_201253	**ENST00000367400**
CRB1	NM_001257965	**ENST00000535699**
CRB1	NM_201253	**ENST00000367400**
CRB1	NM_001257965	**ENST00000535699**
CRB1	NM_201253	**ENST00000367400**
CRB1	NM_001257965	**ENST00000535699**
CRX	NM_000554	**ENST00000221996**
CRX	NM_000554	**ENST00000221996**
CRX	NM_000554	**ENST00000221996**
CRYBA1	NM_005208	**ENST00000225387**
CSPP1	NM_024790	**ENST00000262210**
CTNNB1	NM_001904	**ENST00000349496**
CWC27	NM_005869	**ENST00000381070**
CWC27	NM_001297645	**ENST00000508024**
CWC27	NM_005869	**ENST00000381070**
CWC27	NM_001297645	**ENST00000508024**
CYP1A1	NM_000499	**ENST00000379727**
CYP4V2	NM_207352	**ENST00000378802**
CYP4V2	NM_207352	**ENST00000378802**
DHDDS	NM_024887	**ENST00000360009**
DHX38	NM_014003	**ENST00000268482**
DRAM2	NM_178454	**ENST00000539140**
EFEMP1	NM_001039348	**ENST00000394555**
ELOVL4	NM_022726	**ENST00000369816**
EXOSC2	NM_014285	**ENST00000372358**
EYS	NM_001292009	**ENST00000370621**

EYS	NM_001142801	**ENST00000393380**
FAM161A	NM_001201543	**ENST00000404929**
FAM161A	NM_001201543	**ENST00000404929**
FLVCR1	NM_014053	**ENST00000366971**
FSCN2	NM_001077182	**ENST00000334850**
FSCN2	NM_001077182	**ENST00000334850**
FZD4	NM_012193.3	**ENST00000531380**
Gene	Transcript ID (RefSeq)	Transcript ID (Ensembl)
GNAT1	NM_144499	**ENST00000232461**
GNAT1	NM_144499	**ENST00000232461**
GNAT1	NM_144499	**ENST00000232461**
GNAT2	NM_005272	**ENST00000351050**
GNAT2	NM_005272	**ENST00000351050**
GNAT2	NM_005272	**ENST00000351050**
GNB3	NM_002075	**ENST00000229264**
GNPTG	NM_032520	**ENST00000204679**
GPR125	NM_145290	**ENST00000334304**
GPR179	NM_001004334	**ENST00000342292**
GPR179	NM_001004334	**ENST00000342292**
GRK1	NM_002929	**ENST00000335678**
GRM6	NM_000843	**ENST00000231188**
GRM6	NM_000843	**ENST00000231188**
GUCA1A	NM_000409	**ENST00000053469**
GUCA1A	NM_000409	**ENST00000053469**
GUCA1B	NM_002098	**ENST00000230361**
GUCA1B	NM_002098	**ENST00000230361**
GUCY2D	NM_000180	**ENST00000254854**
GUCY2D	NM_000180	**ENST00000254854**
HGSNAT	NM_152419	**ENST00000379644**
HK1	NM_033497	**ENST00000404387**
HK1	NM_033496	**ENST00000298649**
HK1	NM_001322365	**ENST00000448642**
HK1	NM_033500	**ENST00000360289**
HK1	NM_000188	**ENST00000359426**
HK1	NM_000180	**ENST00000254854**
HK1	NM_033496	**ENST00000298649**
HK1	NM_001322365	**ENST00000448642**
HK1	NM_033500	**ENST00000360289**
HK1	NM_000188	**ENST00000359426**
IDH3A	NM_005530	**ENST00000299518**
IDH3B	NM_006899	**ENST00000380843**
IDH3B	NM_174855	**ENST00000380851**
IFT140	NM014714	**ENST00000426508**
IFT140	NM014714	**ENST00000426508**
IFT140	NM014714	**ENST00000426508**
IFT172	NM_015662	**ENST00000260570**
IFT43	NM_052873	**ENST00000238628**
IFT43	NM_001255995	**ENST00000556742**
IFT43	NM_001102564	**ENST00000314067**
IMPDH1	NM_000883	**ENST00000338791**
IMPDH1	NM_001102605	**ENST00000354269**
IMPDH1	NM_000883	**ENST00000338791**
IMPDH1	NM_001102605	**ENST00000354269**
IMPG1	NM_001563	**ENST00000369950**
IMPG2	NM_016247	**ENST00000193391**

IMPG2	NM_016247	**ENST00000193391**
INPP5E	NM_019892	**ENST00000371712**
INVS	NM_014425	**ENST00000262457**
IQCB1	NM_001023570	**ENST00000310864**
IQCB1	NM_001023570	**ENST00000310864**
IQCB1	NM_001023570	**ENST00000310864**
ITM2B	NM_021999	**ENST00000378565**
KCNJ13	NM_002242	**ENST00000233826**
KCNJ13	NM_002242	**ENST00000233826**
KCNV2	NM_133497	**ENST00000382082**
KCNV2	NM_133497	**ENST00000382082**
KIAA1549	NM_001164665	**ENST00000422774**
KIF11	NM_004523	**ENST00000260731**
KIF11	NM_004523	**ENST00000260731**
KIF7	NM_198525	**ENST00000394412**
KIZ	NM_018474	**ENST00000246027**
KLHL7	NM_001031710	**ENST00000339077**
KLHL7	NM_001172428	**ENST00000322275**
KRT12	NM_000223	**ENST00000251643**
LAMA1	NM_005559	**ENST00000389658**
LAMA1	NM_005559	**ENST00000389658**
LCA5	NM_181714	**ENST00000392959**
LCA5	NM_181714	**ENST00000392959**
LCA5	NM_181714	**ENST00000392959**
LOXL3	NM_032603	**ENST00000264094**
LRAT	NM_004744	**ENST00000336356**
LRAT	NM_004744	**ENST00000336356**
LRIT3	NM_198506	**ENST00000594814**
LRIT3	NM_198506	**ENST00000594814**
LRP5	NM_002335	**ENST00000294304**
MAK	NM_005906	**ENST00000474039**
MERTK	NM_006343	**ENST00000295408**
MERTK	NM_006343	**ENST00000295408**
MFRP	NM_031433	**ENST00000530681**
MFSD8	NM_152778	**ENST00000296468**
MFSD8	NM_152778	**ENST00000296468**
MPDZ	NM_003829	**ENST00000381022**
MPDZ	NM_001261406	**ENST00000447879**
MPDZ	NM_003829	**ENST00000381022**
MPDZ	NM_001261406	**ENST00000447879**
NDP	NM_000266	**ENST00000378062**
NDP	NM_000266	**ENST00000378062**
NEK2	NM_002497	**ENST00000366999**
NEK2	NM_001204183	**ENST00000366998**
NEUROD1	NM_002500	**ENST00000295108**
NMNAT1	NM_022787	**ENST00000377205**
NMNAT1	NM_001297779	**ENST00000403197**
NMNAT1	NM_022787	**ENST00000377205**
NMNAT1	NM_001297779	**ENST00000403197**
NPHP1	NM_000272	**ENST00000316534**
NPHP3	NM_153240	**ENST00000337331**
NPHP4	NM_015102	**ENST00000378156**
NPHP4	NM_015102	**ENST00000378156**
NR2E3	NM014249	**ENST00000567496**
NR2E3	NM014249	**ENST00000567496**

NR2E3	NM014249	**ENST00000567496**
NR2E3	NM014249	**ENST00000567496**
NR2E3	NM014249	**ENST00000567496**
NRL	NM_006177	**ENST00000397002**
NYX	NM022567	**ENST00000342595**
NYX	NM022567	**ENST00000342595**
OFD1	OFD1_intron9	**ENST00000340096**
OFD1	NM_003611_intron9	**ENST00000340096**
OPN1LW	NM_020061	**ENST00000369951**
OPN1MW	NM_000513	**ENST00000369935**
OPN1SW	NM_001708	**ENST00000249389**
OPN1SW	NM_001708	**ENST00000249389**
OR2W3	NM_001001957	**ENST00000537741**
OTX2	NM_021728	**ENST00000339475**
PAX2	NM_003987	**ENST00000428433**
PAX2	NM_003988	**ENST00000361791**
PCYT1A	NM_005017	**ENST00000292823**
PCYT1A	NM_005017	**ENST00000292823**
PDE6A	NM_000440	**ENST00000255266**
PDE6B	NM_000283	**ENST00000496514**
PDE6B	NM_001350154	**ENST00000461490**
PDE6B	NM_000283	**ENST00000496514**
PDE6B	NM_001350154	**ENST00000461490**
PDE6C	NM_006204	**ENST00000371447**
PDE6C	NM_006204	**ENST00000371447**
PDE6C	NM_006204	**ENST00000371447**
PDE6G	NM_002602	**ENST00000331056**
PDE6G	NM_002602	**ENST00000331056**
PDE6H	NM_006205	**ENST00000266395**
PDE6H	NM_006205	**ENST00000266395**
PDE6H	NM_006205	**ENST00000266395**
PIK3R4	NM_014602	**ENST00000356763**
PITPNM3	NM_031220	**ENST00000262483**
PITPNM3	NM_031220	**ENST00000262483**
POC1B	NM_172240	**ENST00000313546**
POC1B	NM_172240	**ENST00000313546**
POMGNT1	NM_001243766	**ENST00000371992**
POMGNT1	NM_017739	**ENST00000371984**
POMGNT1	NM_001290129	**ENST00000535522**
PRCD	NM_001077620	**ENST00000586148**
PROM1	NM_006017	**ENST00000447510**
PROM1	NM_006017	**ENST00000447510**
PROM1	NM_006017	**ENST00000447510**
PRPF3	NM_004698	**ENST00000324862**
PRPF31	NM_015629	**ENST00000321030**
PRPF4	NM_004697	**ENST00000374198**
PRPF6	NM_012469	**ENST00000266079**
PRPF8	NM_006445	**ENST00000304992**
PRPH2	NM_000322	**ENST00000230381**
PRPH2	NM_000322	**ENST00000230381**
PRPH2	NM_000322	**ENST00000230381**
PRPH2	NM_000322	**ENST00000230381**
PRPH2	NM_000322	**ENST00000230381**
RAB28	NM_001017979	**ENST00000330852**
RAB28	NM_004249	**ENST00000288723**

RAB28	NM_001159601	**ENST00000338176**
RASSF8	NM_001164747	**ENST00000542865**
RASSF8	NM_007211	**ENST00000381352**
RAX2	NM_032753	**ENST00000555978**
RAX2	NM_001319074	**ENST00000555633**
RBP3	NM_002900	**ENST00000224600**
RBP4	NM_006744	**ENST00000371464**
RBP4	NM_001323518	**ENST00000371469**
RCBTB1	NM_018191	**ENST00000378302**
RCBTB1	NM_001352505	**ENST00000546015**
RCBTB1	NM_018191	**ENST00000378302**
RCBTB1	NM_001352505	**ENST00000546015**
RD3	NM_183059	**ENST00000367002**
RDH11	NM_016026	**ENST00000381346**
RDH12	NM_152443	**ENST00000551171**
RDH12	NM_152443	**ENST00000551171**
RDH12	NM_152443	**ENST00000551171**
RDH5	NM_002905	**ENST00000257895**
REEP6	NM_138393	**ENST00000233596**
REEP6	NM_001329556	**ENST00000395479**
RGR	NM_002921	**ENST00000359452**
RHO	NM_000539	**ENST00000296271**
RHO	NM_000539	**ENST00000296271**
RIMS1	NM_014989	**ENST00000521978**
RIMS1	NM_001168407	**ENST00000401910**
RIMS1	NM_001168410	**ENST00000517827**
RIMS1	NM_014989	**ENST00000521978**
RIMS1	NM_001168407	**ENST00000401910**
RIMS1	NM_001168410	**ENST00000517827**
RIMS2	NM_001100117	**ENST00000406091**
RIMS2	NM_014677	**ENST00000507740**
RIMS2	NM_001282882	**ENST00000339750**
RIMS2	NM_001282881	**ENST00000262231**
RIMS2	NM_001348509	**ENST00000408894**
RIMS2	NM_001100117	**ENST00000406091**
RIMS2	NM_014677	**ENST00000507740**
RIMS2	NM_001282882	**ENST00000339750**
RIMS2	NM_001282881	**ENST00000262231**
RIMS2	NM_001348509	**ENST00000408894**
RLBP1	NM_000326	**ENST00000268125**
RLBP1	NM_000326	**ENST00000268125**
ROM1	NM_000327	**ENST00000278833**
RP1	NM_006269	**ENST00000220676**
RP1L1	NM_178857	**ENST00000382483**
RP1L1	NM_178857	**ENST00000382483**
RP2	NM_006915	**ENST00000218340**
RP9	NM_203288	**ENST00000297157**
RPE65	NM_000329	**ENST00000262340**
RPE65	NM_000329	**ENST00000262340**
RPE65	NM_000329	**ENST00000262340**
RPGR	NM_001034853	**ENST00000378505**
RPGR	NM_000328	**ENST00000318842**
RPGR	NM_001034853	**ENST00000378505**
RPGR	NM_000328	**ENST00000318842**
RPGR	NM_001034853	**ENST00000378505**

RPGR	NM_000328	**ENST00000318842**
RPGRIP1	NM_020366	**ENST00000400017**
RPGRIP1	NM_020366	**ENST00000400017**
RPGRIP1	NM_020366	**ENST00000400017**
RPGRIP1L	NM_015272	**ENST00000379925**
RPGRIP1L	NM_001328423	**ENST00000566096**
RPGRIP1L	NM_001328422	**ENST00000568653**
RS1	NM_000330	**ENST00000379984**
SAG	NM_000541	**ENST00000409110**
SAG	NM_000541	**ENST00000409110**
SDCCAG8	NM_006642	**ENST00000366541**
SDCCAG8	NM_001350248	**ENST00000476722**
SEMA4A	NM_022367	**ENST00000368285**
SEMA4A	NM_022367	**ENST00000368285**
SLC24A1	NM_004727	**ENST00000261892**
SLC24A1	NM_001301033	**ENST00000537259**
SLC24A1	NM_004727	**ENST00000261892**
SLC24A1	NM_001301033	**ENST00000537259**
SLC24A1	NM_004727	**ENST00000261892**
SLC24A1	NM_001301033	**ENST00000537259**
SLC38A8	NM_001080442	**ENST00000299709**
SLC7A14	NM_020949	**ENST00000231706**
SNRNP200	NM_014014	**ENST00000323853**
SPATA7	NM_018418	**ENST00000393545**
SPATA7	NM_018418	**ENST00000393545**
SPATA7	NM_018418	**ENST00000393545**
SRD5A3	NM_024592	**ENST00000264228**
SRD5A3	NM_024592	**ENST00000264228**
TIMP3	NM_000362	**ENST00000266085**
TMEM138	NM_016464	**ENST00000278826**
TMEM216	NM_001173991	**ENST00000334888**
TMEM237	NM_001044385	**ENST00000409883**
TMEM237	NM_152388	**ENST00000409444**
TOPORS	NM_005802	**ENST00000360538**
TRPM1	NM_001252020	**ENST00000542188**
TRPM1	NM_001252024	**ENST00000256552**
TRPM1	NM_001252030	**ENST00000559179**
TRPM1	NM_001252020	**ENST00000542188**
TRPM1	NM_001252024	**ENST00000256552**
TRPM1	NM_001252030	**ENST00000559179**
TSPAN12	NM_012338	**ENST00000222747**
TTC8	NM_001288781	**ENST00000338104**
TTC8	NM_001288782	**ENST00000358622**
TTC8	NM_144596	**ENST00000380656**
TTC8	NM_001288781	**ENST00000338104**
TTC8	NM_001288782	**ENST00000358622**
TTC8	NM_144596	**ENST00000380656**
TTLL5	NM_015072	**ENST00000298832**
TTLL5	NM_015072	**ENST00000298832**
TTPA	NM_000370	**ENST00000260116**
TUB	NM_003320	**ENST00000305253**
TUB	NM_177972	**ENST00000299506**
TUBB4B	NM_006088	**ENST00000340384**
TULP1	NM_003322	**ENST00000229771**
TULP1	NM_003322	**ENST00000229771**

TULP1	NM_003322	**ENST00000229771**
UNC119	NM_005148	**ENST00000335765**
UNC119	NM_054035	**ENST00000301032**
USH2A	NM_206933	**ENST00000307340**
USH2A	NM_007123	**ENST00000366942**
USH2A	NM_206933	**ENST00000307340**
USH2A	NM_007123	**ENST00000366942**
VCAN	NM_004385	**ENST00000265077**
VPS13B	NM_017890	**ENST00000358544**
VPS13B	NM_152564	**NST00000357162**
VPS13B	NM_181661	**ENST00000441350**
VPS13B	NM_015243	**ENST00000355155**
WDR19	NM_025132	**ENST00000399820**
WDR19	NM_025132	**ENST00000399820**
ZNF408	NM_024741	**ENST00000311764**
ZNF408	NM_024741	**ENST00000311764**
ZNF423	NM_015069	**ENST00000561648**
ZNF513	NM_144631	**ENST00000323703**
