# Supplementary material for: Four Unique Genetic Variants in Three Genes Account for 62.7% of Early-Onset Severe Retinal Dystrophy in Chile: Diagnostic and Therapeutic Consequences
Source: Int J Mol Sci. 2024 Jun 3;25(11):6151. doi: 10.3390/ijms25116151 (PMC11172861; doi:10.3390/ijms25116151)
Supplement: Supplementary file 1 [file ijms-25-06151-s001.zip › Tables/Supplementary Tables S2.pdf]

Gene	Variant	Exon	Primers	
			Forward 5'-3'	Reverse5'-3'
ALMS1	NM_001378454.1: c.1092del/p.(Asp365Ilefs*11)	5	ACCATCGGAAGTTAGTGAAGCT	CCTGTGTCCAATACTGCCCA
CEP290	NM_025114.3:c.38T>A/p.(Val13Asp)	2	AATGCTAATATACACCTGTTT	AGATTTTTATAGTTCCACTAAT
CRB1	NM_201253.2:c.653-1G>A	Intron 2	GCTCTGGTAAACAAAGCATTG	GAATCCAGGGGCACAGTCG
CRB1	NM_201253.2:c.750T>A/p.(Cys250*)	3	GACGAATGTTGGTCCCAGC	CAGAGTGGTAAAATAGTTCATG
CRB1	NM_201253.2:c.798_799del/p.(Ala267Glnfs*18)	3		
CRB1	NM_201253.2:c.2291G>A, p.(Arg764His)	7	CTCCATGTTTGTCCGAACGC	TCTTGCTTGTCAGGTAGGC
CRB1	NM_201253.2:c.2843G>A/p.(Cys948Tyr)	9	AATGATCATTACTATTAATAACGG	GTGCCATCATTCACTGACTG
CRB1	NM_201253.2:c.3110_3143dup/p.(Ser1049Aspfs*40)	9	GTGGCAACAGCTTTTATATGC	CATGAACATTTTCAAAGTAAGAG
GUCY2D	NM_000180.3:c.389del/p.(Pro130Leufs*36)	2	GGTCCCCGCTTCGAGGTAG	GAGTGCCGTGGACAGTGAG
GUCY2D	NM_000180.3:c.1343C>A/p.(Ser448*)	4	GTGGGCTGTGACCCCGACCT	TGGTCCATGGCGATTGTCTC
LCA5	NM_181714.3:c.1243G>T/p.(Glu415*)	9	ACTGGAAAGAGAAGAAAAGCCA	TGGATTTGACCTCTCTGATGTT
NMNAT1	NM_001297778.1:c.364del/p.(Arg122Glyfs*20)	4	TGTGAGCCACTGTGCCCAGC	ACTGCACTCCAGCCTGGGTG
NMNAT1	NM_001297778.1:c.507G>A/(p.Trp169*)	5	TTATTCTTCCCAGCTGTGCC	CCCAGATTGTTTCAGATCCCC
NMNAT1	NM_001297778.1:c.769G>A/p.(Glu257Lys)	5		
RAB28	NM_001017979.3:c.331_333del/p.(Val111del)	4	CCACCTCCAGACACCATCAT	CCCATTTTGACATATCTGAAGCA
RDH12	NM_152443.2:c.295C>A/p.(Leu99Ile)	5	CCAGTCCCAAGCTCACTTAC	AGGGTGGAGCAGCCACTC
RDH12	NM_152443.2:c.716G>T/p.(Arg239Leu)	8	TGTGTATTTTGCTGCAGGAG	GATGAACAGCCCAGCGAG
RP1	NM_006269.1:c.5564del/p.(Lys1855Argfs*42)	4	GGAACTGACTCAACCCCTTG	CGGTCTTCCTCATTCATGGGT
TULP1	NM_003322.6:c.1149C>A/p.(Asp383Glu)	12	GGATGTAGGATCCCCTCAGC	GTTTCTCACATAGGGAGCCAGG
NR2E3	NM_014249.4:c.932G>A/(p.Arg311Gln)	6	GTGCCTGAGATGGTGGCAGA	GGGAGAGTGAGAGGCAGA
